# Meaningful Interpretation of Subdiffusive Measurements in Living Cells (Crowded Environment) by Fluorescence Fluctuation Microscopy

**DOI:** 10.2174/138920110791591454

**Published:** 2010-08

**Authors:** Gerd Baumann, Robert F Place, Zeno Földes-Papp

**Affiliations:** 1 Mathematics Department, German University in Cairo;; 2 Helen Diller Comprehensive Cancer Center, University of California, San Francisco, CA, USA;; 3 Department of Urology, University of California, San Francisco, CA, USA;; 4 Medical University of Graz, Austria

**Keywords:** Anomalous motion, broken ergodicity, Continuous Time Random Walks (CTRW), Continuous Time Random Walks (CTRW) on fractal supports, cellular crowding, Cytoplasmic Assembly of Nuclear RISC, ergodicity, FCS, FCCS, Fluorescence Fluctuation Microscopy, GFP-Ago1, GFP-Ago2, heterogeneity, living cells, meaningful interpretation of subdiffusive measurements, microRNA trafficking, physical model of crowding, physical model of heterogeneity, random walks on fractal supports, resolution limits of measured diffusion times for two components, RNA Activation (RNAa), Single Molecule, Small Activating RNA (saRNA), Temporal autocorrelation, Temporal two-color crosscorrelation, Fluorescence imaging, Time dependence of apparent diffusion coefficients.

## Abstract

In living cell or its nucleus, the motions of molecules are complicated due to the large crowding and expected heterogeneity of the intracellular environment. Randomness in cellular systems can be either spatial (anomalous) or temporal (heterogeneous). In order to separate both processes, we introduce anomalous random walks on fractals that represented crowded environments. We report the use of numerical simulation and experimental data of single-molecule detection by fluorescence fluctuation microscopy for detecting resolution limits of different mobile fractions in crowded environment of living cells. We simulate the time scale behavior of diffusion times τ_D_(τ) for one component, e.g. the fast mobile fraction, and a second component, e.g. the slow mobile fraction. The less the anomalous exponent α the higher the geometric crowding of the underlying structure of motion that is quantified by the ratio of the Hausdorff dimension and the walk exponent *d _f_* /*d_w_* and specific for the type of crowding generator used. The simulated diffusion time decreases for smaller values of α ≠ 1 but increases for a larger time scale τ at a given value of α ≠ 1. The effect of translational anomalous motion is substantially greater if α differs much from 1. An α value close to 1 contributes little to the time dependence of subdiffusive motions. Thus, quantitative determination of molecular weights from measured diffusion times and apparent diffusion coefficients, respectively, in temporal auto- and crosscorrelation analyses and from time-dependent fluorescence imaging data are difficult to interpret and biased in crowded environments of living cells and their cellular compartments; anomalous dynamics on different time scales τ must be coupled with the quantitative analysis of how experimental parameters change with predictions from simulated subdiffusive dynamics of molecular motions and mechanistic models. We first demonstrate that the crowding exponent α also determines the resolution of differences in diffusion times between two components in addition to photophyscial parameters well-known for normal motion in dilute solution. The resolution limit between two different kinds of single molecule species is also analyzed under translational anomalous motion with broken ergodicity. We apply our theoretical predictions of diffusion times and lower limits for the time resolution of two components to fluorescence images in human prostate cancer cells transfected with GFP-Ago2 and GFP-Ago1. In order to mimic heterogeneous behavior in crowded environments of living cells, we need to introduce so-called continuous time random walks (CTRW). CTRWs were originally performed on regular lattice. This purely stochastic molecule behavior leads to subdiffusive motion with broken ergodicity in our simulations. For the first time, we are able to quantitatively differentiate between anomalous motion without broken ergodicity and anomalous motion with broken ergodicity in time-dependent fluorescence microscopy data sets of living cells. Since the experimental conditions to measure a selfsame molecule over an extended period of time, at which biology is taken place, in living cells or even in dilute solution are very restrictive, we need to perform the time average over a subpopulation of different single molecules of the same kind. For time averages over subpopulations of single molecules, the temporal auto- and crosscorrelation functions are first found. Knowing the crowding parameter α for the cell type and cellular compartment type, respectively, the heterogeneous parameter γ can be obtained from the measurements in the presence of the interacting reaction partner, e.g. ligand, with the same α value. The product $\alpha \cdot \gamma =\tilde{\gamma }$ is not a simple fitting parameter in the temporal auto- and two-color crosscorrelation functions because it is related to the proper physical models of anomalous (spatial) and heterogeneous (temporal) randomness in cellular systems. We have already derived an analytical solution for $\tilde{\gamma }$ in the special case of *γ* = 3/2 . In the case of two-color crosscorrelation or/and two-color fluorescence imaging (co-localization experiments), the second component is also a two-color species *gr*, for example a different molecular complex with an additional ligand. Here, we first show that plausible biological mechanisms from FCS/ FCCS and fluorescence imaging in living cells are highly questionable without proper quantitative physical models of subdiffusive motion and temporal randomness. At best, such quantitative FCS/ FCCS and fluorescence imaging data are difficult to interpret under crowding and heterogeneous conditions. It is challenging to translate proper physical models of anomalous (spatial) and heterogeneous (temporal) randomness in living cells and their cellular compartments like the nucleus into biological models of the cell biological process under study testable by single-molecule approaches. Otherwise, quantitative FCS/FCCS and fluorescence imaging measurements in living cells are not well described and cannot be interpreted in a meaningful way.

## 1 INTRODUCTION

In living cells, the abundance of biomacromolecules in the cytoplasm and nucleus causes a state of molecular crowding [1-4]. Translational motion in crowded environment is not well represented by normal, Brownian diffusion [5]. Translational diffusive measurements by fluorescence fluctuation microscopy (FFM) like fluorescence correlation spectroscopy (FCS) are difficult to interpret in a crowded environment of a living cell because conventional analysis of fluorescence fluctuation time series such as auto- and crosscorrelation function assumes that the spatial domain available for diffusive motion is unlimited [6]. This assumption is invalid for a crowded environment of a biological cell. The overall translational motion of molecules in living cells is better described by distances between collisions that are not independent and can take different times to perform. The temporal autocorrelation and the temporal crosscorrelation of the signal with itself reveals the time required for a molecule to diffusion through an open observation volume ΔV. Diffusive motion is the macroscopic result of random thermal motions that occur on a molecular level, and the collision between solute particles/molecules and solvent molecules experiencing thermal movement are responsible for this phenomenon. Along this line, translational anomalous diffusion is interesting to explore because it is a prototype of subdiffusion in biological cells and their compartments like membranes [7]. A common way to study deviations from normal Gaussian diffusion is to plot the mean square displacement (MSD) of the molecules and particles, respectively, in *n*-dimensional space. In systems, where molecules move freely via 3D Brownian dynamics, the MSD is proportional to time and given by $\langle {\vec{r}}^{2}\left(t\right)\rangle =6\cdot D\cdot t$. *D *and *t *are the diffusion coefficient and time. Any deviation from linearity corresponds to translational anomalous diffusion [8] with $\langle {\vec{r}}^{2}\left(t\right)\rangle =\Gamma_{\alpha }\cdot t^{\alpha }$ and α ≠ 1. We focus on subdiffusion, i.e. 0 < α < 1. While the MSD is used to classify a process as subdiffusion, it does not provide any information on the physical and biological mechanism underlying the subdiffusion. A physical correct propagator for subdiffusive temporal evolution based on the instantaneous diffusion coefficient $D_{\mathrm{inst}}\left(t\right)=\frac{\alpha }{6}\cdot \Gamma \cdot t^{\alpha -1}$ was first described, producing the correct power law scaling of MSD versus time and rigorously solved the extended diffusion equation from Fick's law and the continuity equation [9]. With that propagator, the correct FCS autocorrelation function for translational anomalous diffusion was derived [9], where the temporal autocorrelation defines the time scale τ of diffusion with the characteristic diffusion time τ_D_ across the 3D observation volume ΔV for *m*-photon excitation and *n*-dimensional motion as

(1) $$\tau_{D}={\left(\frac{\omega_{x-y}^{2}}{4\cdot m\cdot \Gamma /2\cdot n}\right)}^{1/\alpha }=\left(\frac{\omega_{x-y}^{2}}{4\cdot m\cdot D_{\mathrm{app}}\left(\tau \right)}\right)=\left(\frac{{\alpha \cdot \omega }_{x-y}^{2}}{4\cdot m\cdot D_{\mathrm{inst}}\left(\tau \right)}\right)\cdot$$

*ω*_x-y_ is the radial waist of excitation laser and *D*_app_ the measured or apparent diffusion coefficient, e.g. in FCS. With this clarification, it becomes more feasible to unambiguously report mobility data in terms of either a well-defined diffusion time τ_D_ or as time-dependent diffusion coefficients [9]. Two-color crosscorrelation analysis (FCCS) for one-photon excitation only requires in Eqn. (1) that

(2) $$\tau_{D,\mathrm{gr}}=\frac{\tau_{D,g}+\tau_{D,r}}{2}=\frac{\omega_{x-y,g}^{2}+\omega_{x-y,r}^{2}}{8D_{\mathrm{app}}\left(\tau \right)}$$

where *ω_x-y,g_* and *ω_x-y,r_* are the radial waists of the green (or blue) and red excitation lasers for the measurement of the two-color labeled molecular species *gr*. The most commonly published form of the time-dependent 3D diffusion coefficient is defined as [7]

(3) $$D_{\mathrm{app}}\left(t\right)=\frac{\Gamma }{6}\cdot t^{\alpha -1}$$

such that the MSD can then be written as

(4) $$\langle {\vec{r}}^{2}\left(\tau \right)\rangle =6\cdot D_{\mathrm{app}}\left(\tau \right)\cdot \tau .$$

The apparent 3D diffusion coefficient *D*_app_(*τ*) is found at time *τ* from the slope of a straight line between the origin and MSD(*τ*) in the linear plot of MSD(*τ*) versus *τ* [9]. Using FFM and FCS to investigate translational anomalous mobility in living cells requires a through understanding of the theoretical basis of fitting models and the physical significance of associated fitting parameters as we shall demonstrate in this original article.

In FFM, movement of fluorescent molecules through subfemtoliter observation volume ΔV generates temporal fluctuations in the fluorescence emitted across the 3D observation volume. We demonstrated by simulations and experiments that at the single-molecule level there is only one molecule at a time inside ΔV or no molecule in dilute solution-phase [10]. With the average molecule number N = 0.0055 for ΔV = 0.21 fL measured with 24-nm nanospheres at 635 nm laser excitation and N = 0.0052 for ΔV = 0.14 fL measured with 100-nm nanospheres at 470 nm laser excitation in the real fluorescence fluctuation time series, we reached the lower single-molecule detection limit for dilute solutions. The contribution of two simultaneously fluorescing molecules to the detected signal during the measurement vanished for N << 1 [10], and the single-molecule detection regime came close to the average N = 0.048 and N = 0.0057 molecules and particles, respectively, per ΔV [11]. In this paper, we analyze translational anomalous, subdiffusive motion without broken ergodicity and with broken ergodicity in the 3D-measurement set that includes an ensemble average of single molecules, or a single molecule, an observation volume ΔV and a local crowded or/and heterogeneous environment. The article aims to remove some wide-spread confusion regarding the interpretation of subdiffusive measurements in crowded and heterogeneous environment of living cells by time-dependent fluorescence fluctuation microscopy.

We apply our theoretical predictions of diffusion times and lower limits for the time resolution of two components to fluorescence images in human prostate cancer cells (PC-3) transfected with fusion proteins of green fluorescence protein (GFP) and argonaute proteins (Ago1, Ago2). Experiments in living cells and their compartments are challenged by the fact that diffusion is slower and heterogeneous compared to *in vitro* systems of dilute solutions [6]. The cell response depends on a spatially organized system of biomacro-molecules like proteins, RNAs, DNA. To sustain cellular activity, the spatial crowding of biomacromolecules is maintained within the intact cellular architecture [12-14]. We first address the problem how translational anomalous, subdiffusive motion dominates the time-dependent behavior of an ensemble average of single molecules or a single molecule. We shall show that ignoring the observed effects of crowding and heterogeneity may produce results that cannot be interpreted in a meaningful way.

## 2 METHODS

### 2.1 Random Walks on Fractals

There is a pronounced interest in clarifying the behavior of random walks (RW) on fractal supports [15]. The approaches that we use are random walks on a fractal support and continuous time random walks (CTRW) [8, 16]. Both approaches aim on the clarification of the interrelation of random processes connected with real diffusion phenomena observed in experiments. The majority of work found in literature, however, is restricted to one dimensional or two dimensional systems [8, 17]. Only a few articles deal with the real three dimensional problem [15]. In uniform Euclidean systems, the mean-square displacement $\langle {\vec{r}}^{2}\rangle$ of a random walker is proportional to the time *t* for any number of spatial dimensions *d* . However, in disordered systems, this law is not valid in general, and the diffusive law becomes anomalous [18, 19]

(5) $$\langle {\vec{r}}^{2}\rangle =\Gamma_{\alpha }\cdot t^{\alpha },$$

which in addition with the first moment, the mean, is discussed as a finger print of normal (α = 1) and anomalous ( 0 < *α* <1) diffusion [17]. It is well known that diffusive motion on a fractal support is subdiffusive with the MSD in time *t* given by Eqn. (5). The exponent α is related to the fractal dimension of the walker *d_w_* = 2/*α* [20]. The slowing down of the motion is caused by the delay of the diffusing molecules in the dangling ends, bottlenecks and backbends existing in the disordered structure. On the other hand, Eqn. (5) is based on the existence of the term $\langle {\vec{r}}^{2}\rangle$, which is for an ergodic system either the ensemble mean over all random trajectories or the time average of a single trajectory [8]. Thus, the first approach in this article is to examine the motion on a fractal support as an ensemble of tracks carried out on it. Two and three dimensional examples of tracks of random walk on fractal support are shown in Fig. (**1**). In a simple discrete random walk, the walker advances one step in unit time. Each step is taken to a nearest neighbor of the site. The simple random walker can step with equal probability to any of the nearest neighbor sites that belongs to the grid. There are several ways of assigning transition probabilities for stepping from site to site [15]. The motion of the Brownian molecule is generated with a random number generator delivering pseudo-random numbers used for the steps in all three spatial directions. We generate a random Brownian walk by randomly selecting steps in the three coordinate directions. The three coordinate directions are generated by a permutation of the vector $\vec{v}=\left(0,0,1\right)$ so that a set of orthogonal vectors S is generated. Mathematically this means we use the basic set of orthogonal unit vectors in a Cartesian coordinate system as the basis of our calculations

(6) $$S=\left\{\vec{i},\vec{j},\vec{k}|\vec{i},\vec{j},\vec{k}\varepsilon {\mathbb{R}}^{3}ˆ\left\|\vec{i}\right\|=1,\left\|\vec{j}\right\|=1,\left\|\vec{k}\right\|=1\right\}.$$

This set of permuted vectors is extended in all directions positive and negative by the following unification of basis sets

(7) $$S^{*}=S\cup -S=\left\{\vec{i},\vec{j},\vec{k},-\vec{i},-\vec{j},-\vec{k}\right\}.$$

Introducing the random function R*_k_* which selects the direction with equal probability randomly from our basis set S^*^, we create the Brownian track $B_{n}\left({\vec{r}}_{0},\vec{r}\right)$ by a sum of independent vectors

(8) $$B_{\mathrm{track}}:={\vec{r}}_{0}+\sum_{k=1}^{n}R_{k}\left(S^{*}\right)=B_{n}\left({\vec{r}}_{0},\vec{r}\right),$$

A simple random walk is statistically self-similar [15, 8]. The random steps are distributed according to a probability function $P_{n}\left(\vec{r}\right)$. In the limit that n >> 1, $P_{n}\left(\vec{r}\right)$ tends to a Gaussian distribution. This is a simple result of the Central-Limit Theorem. A direct consequence of the self-similarity is that the simple random walk is a statistical fractal. Upon dilation of space by a factor of *λ*^1/2^, the number of steps increases by a factor of *λ*. Therefore, the fractal dimension of a random walk is *d_w_* = ln(*λ*)/ln(*λ*^1/2^)=2 . It is a curious result that random walks performed on disordered but statistically self-similar structures are still self-similar themselves, exactly as in Euclidean space. The quantitative difference is that the usual diffusion exponent, *d_w_* = 2 is no longer equal to 2 and diffusion becomes anomalous. In order to generate an anomalous random walk, we use the set S^*^ and the random function R*_k_* restricted to a fractal structure which is produced on a virtual grid. Examples of such tracks are shown in Fig. (**1**) for two and three dimensions. This approach can be easily generalized to higher dimensions if the basis set S is generalized to the corresponding span of the space under consideration. How the fractal support is generated will be discussed below.

The finger print of RW is its mean square displacement. The MSD on a 3D random walk on fractal support is compared with the 2D-RW in Fig. (**2**). The MSD in all cases is determined by the ensemble average of *M* tracks of length *n* using the random track $B_{n}\left({\vec{r}}_{0},\vec{r}\right)$

(9) $${\langle {\vec{r}}^{2}\rangle }_{\mathrm{es}}=\frac{1}{M}\sum_{k=1}^{M}\left(B_{n}\left({\vec{r}}_{0},\overset{\Rightarrow }{r_{0}}\right)-B_{n}\left({\vec{r}}_{0},{\overset{\leftarrow }{r}}_{E}\right)\right)\cdot \left(B_{n}\left({\vec{r}}_{0},{\vec{r}}_{0}\right)-B_{n}\left({\vec{r}}_{0},{\vec{r}}_{E}\right)\right).$$

If ergodicity is given an equivalent representation can be formulated for the MSD, a moving average over a single trajectory of length *n*

(10) $${\langle {\vec{r}}^{2}\rangle }_{T}=\frac{1}{T-\Delta }\int_{0}^{T-\Delta }\left(\vec{B}\left(t+\Delta \right)-\vec{B}\left(t\right)\right)\cdot \left(\vec{B}\left(t+\Delta \right)-\vec{B}\left(t\right)\right)\mathrm{dt},$$

where $\vec{B}\left(t\right)$ is the continuous representation of $B_{n}\left({\vec{r}}_{0},\vec{r}\right)$ and Δ is the lag time for the continuous random walk of length *T*. *T* is the measurement time in the real experiment.

### 2.2 Generation of Fractals

The term fractal was introduced by Mandelbrot [21] and is nowadays used to distinguish irregular structures in nature that are self-similar on certain length scales [22]. Fractals are showing up almost everywhere, for examples as coastlines, cells, human lungs, or percolation clusters [23]. The mentioned examples are so called statistical fractals that, in contrary to deterministic fractals, show the self-similar behavior only on a limited length scale [23-27]. However, deterministic fractals show a self-similar behavior on all length scales. One of the most prominent examples of a deterministic fractal is the Sierpinski gasket in 2D [8] and the Sierpinski carpet (Menger sponge) in 3D. To construct the Sierpinski gasket we start with a square which is divided into nine equal subsquares, where the central square is removed. This first step of the construction is called the generator of the 2D Sierpinski gasket. If we continue with the construction in the same way for each of the remaining subsquares the second iteration step of the fractal is generated [8]. Continuing to infinity, we will get a structure with a self-similar behavior on all scales containing many holes arranged in a symmetric way. The same procedure can be used to construct the 3D version of the Sierpinski gasket which is also self-similar and has the appearance of a sponge. However, the 2D and 3D construction are not restricted to this simple pattern. In fact, it is possible by the same method to generalize the Sierpinski gasket and the carpet to a different structure, if we not only delete one element in the generator but, instead, allow the deletion of more than one element. This, of course, results into a great variety of generalized Sierpinski patterns introducing a variation of the gasket and the sponge. In our examinations, we will restrict us to generalized Sierpinski carpets (GSC), which delete not more than half of the elements of the generator in *d* dimensions.

We begin by considering a class of fractal subsets of R*^d^* formed by the following generalization of the construction of the Cantor ternary set. Let *d*≥2 and let S_0_ = [0,1]^*d*^ = [0,1]×[0,1]×...×[0,1] the *d*-dimensional Cartesian product. Let *ν* ≥ 3 be an integer and divide *S*_0_ into *ν_d_* equal subcubes. Next remove a symmetric pattern with o elements of subcubes from *S*_0_ and call what remains *S*_1_. Now repeat the procedure: divide each subcube that is contained in *S*_1_ into *ν_d_* equal parts, remove the same symmetric pattern from each as was done to obtain *S*_1_ from *S*_0_, and call what remains *S*_2_. Continuing in this way we obtain a decreasing sequence of (closed) subsets of [0,1]^*d*^ . Let $S=\overset{\infty }{\underset{n=0}{\cap }}S_{n}$ we call *S* a generalized Sierpinski carpet (GSC) or simply, a carpet. We may regard the set *S* as idealized models of a region with diffusion obstacles of many different sizes. The standard SC is the GSC for which *d* = 2 , *ν* = 3 , and *S*_1_ consists of *S*_0_ minus the central square. Let *µ = ν*d* -o* be the number of subcubes remaining in *S*_1_ , and let

(11) $$d_{f}=\ln\left(\mu \right)/\ln\left(\nu \right).$$

Hence, the Hausdorff dimension of *S* is *d_f_*. An example of a GSC in ${\mathbb{R}}^{3}$, is depicted in Fig. (**3**), where *d* = 3 and *ν* = 3 with µ = 15.

This generation of a fractal is based on the renormalization of the whole structure and can be used efficiently to generate a fractal support on an infinite space. Since *µ* < *ν*^3^ for *d* = 3 is the number of occupied sites that can be arranged on *ν*^3^ sites in an independent way, a large number of arrangements is possible. In fact, if we count all the different arrangement we will find $\rho =\left(\begin{array}{c} \nu^{3}\\ \mu \end{array}\right)$ arrangements; e.g. *µ* = 22 and *ν* = 3 allows *ρ* = 80730 possible patterns. This means that the arrangement of the occupied cubes determine the various patterns, which can be created each having the same Hausdorff dimension *d_f_* . However, the partition of the space will be different for each of the unique generators. It becomes obvious due to the binomial behavior of arrangements that there exist a large variety of generators possessing all the same dimension *d_f_* .

After discussing how fractals are generated on latices, we perform random walks on these lattices. As these fractals have holes on every length scale due to their construction procedure, the diffusion on such structures is slowed down

(12) $$\langle {\vec{r}}^{2}\rangle =\Gamma_{\alpha }\cdot t^{\alpha }=\Gamma_{\alpha }\cdot t^{2/d_{w}}.$$

The walk exponent *d_w_* is usually greater than two for fractals. The relation *α* = 2/*d_w_* < 1 is valid for any Euclidean dimension. For a random walk on a fractal support, *t* is equivalent to the number of steps *n*.

A random walk (RW) along the occupied sites gives the walk exponent *d_w_* from Eqn. (12) [28]. The value of *d_w_* is determined not only by *d_f_* but also by other details of the generator. The deviation between *d_w_* and *d_f_* are discussed using the term lacunarity to explain the differences [21, 28, 29]. A relation between the Hausdorff dimension and the walk exponent using the equivalence of the vibration and diffusion problem was derived by Alexander and Orbach [18]

(13) $$\frac{d_{f}}{d_{w}}=\frac{d_{s}}{2},$$

where *d_s_* is the spectral dimension. The great interest in the conjecture results not only from the universal value assigned to *d_s_* = 4/3, but also from the fact that it provides a relationship between *dynamic *and *static *exponents. Indeed, by assuming *d_s_* = 4/3 = constant in the exact relation, one immediately obtains *d_w_* = 2*d_f_*/*d_s_*, characterizing the *dynamic* process of diffusive motion, in terms of *d_f_*, which describes the *static *geometrical scaling of the mass of the molecule. While the exact determination of *d_s_* seems to be a hard problem, it is quite easy to obtain certain bounds, and we have in particular that 1 < *d_s_* ≤ *d_f_* < *d* .

The majority of random walk algorithms in use for RW on fractal supports have huge memory requirements, as a complete fractal with a fixed iteration depth is stored. In our algorithm, we do not store a generated pattern of the fractal up to some definite stage [8, 17]. We only supply the generator as input. The random walker is set on some site and it tests whether each site it arrives at is an allowed site, as it goes along. This kind of walk generation is known as the blind ant approach [30]. The actual procedure is as follows: The walk can start at any site of the underlying virtual lattice. To check whether a site is accessible, the first step is to identify the iteration stage the point belongs to. For an *ν*^3^ grid, a point having either an *x^(i)^*-coordinate (*i* =1,2,3) between *ν*^*k*-1^ and *ν^k^* belongs to the *k* th iteration stage of the fractal. In the *k* th-stage coarse-grained pattern with units of size *ν*^*k*-1^, it is checked whether the block containing the site matches an accessible site on the given generator. If found accessible, the corresponding point in the next lower stage, i.e. (*k* - 1) , is ascertained. In this way, the point is successively scaled down until it reaches the first stage. In general, in the *k* th stage, the equivalent coordinates $\left(x_{k}^{\left(1\right)},x_{k}^{\left(2\right)},x_{k}^{\left(3\right)}\right)$ are given by the integer parts of $x_{k}^{\left(i\right)}/\nu^{k-1}$ with *i* =1,2,3 . If $\left(x_{k}^{\left(1\right)},x_{k}^{\left(2\right)},x_{k}^{\left(3\right)}\right)$ matches an allowed site, the coordinates carried over to the next stage are $x_{k-1}^{\left(i\right)}=\mathrm{mod}\left(x_{k}^{\left(i\right)}\cdot \nu^{k-1}\right)$; *i* =1,2,3 . If an equivalent coordinate of any stage does not match the list of accessible sites, the site under consideration is blocked, only those sites surviving up to stage 1 are accessible. If the point corresponds to a blocked site, at any stage of the process, it is inaccessible. This procedure of coarse graining the grid corresponds to a renormalization of the lattice [15]. It is useful to know conditions under which scaling exponents α and values of $\Gamma_{\alpha }$ are generated through the random walk on the 3D Sierpinski carpet. As exemplified in Table **1**, α and $\Gamma_{\alpha }$ are a consequence of the chosen generator of the 3D Sierpinski carpet.

### 2.3 3D Measurement Set

**Fig. (1):**
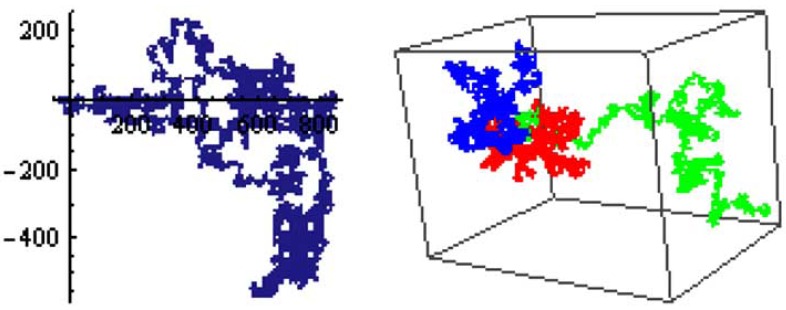
Representation of fractal tracks in two and three dimensions. Left: a track on a Sierpinski gasket. Right: three different trajectories on a generalized 3D Sierpinski carpet. The left panel shows that the 2D random walk on fractal support is traversing the fractal geometry densely in some parts of the fractal. The right panel shows that a 3D random walk on the fractal support is examining the space on the fractal, however the fractal support is not obvious for these short tracks with a total number of steps *n* =10^4^ .

This original research article deals with a 3D measurement set which includes an ensemble of single molecules, or a molecule, an observation volume ΔV and a local environment [10]; in contrast to our previous paper [10], in this article the local environment is the crowded and heterogeneous environment like a biological cytoplasm or the cellular compartment of a nucleus and not a dilute solution. As explained above and depicted in Fig. 1, single-molecules show translational anomalous diffusive motion, which we simulate by fractal dynamic behavior of the 3D random Brownian movements. In fluorescence fluctuation microscopy, no information can be obtained about the single molecules as long as the molecule dwells outside the 3D observation volume ΔV. The dynamics of the molecule remains hidden unless it is the dynamics of the molecule itself that causes the change in the molecule number fluctuations across ΔV. For single-molecule fluctuation counting, we introduced the scalar function *η*(t) for the real experimental situation of measuring a single molecule at a time [10]

(14) $$\eta \left(t\right)=\left\{\begin{array}{cc} 1 & \mathrm{inside \Delta V,emitted fluorescence detected,}\\ 0 & \mathrm{outside\Delta V,no florescence detected.} \end{array}\right.$$

Consequently, there is a simple in and out jump of the molecule or an on and off of the signal constrained by ΔV as shown in Figs. (**2** and **4**). This represents the real molecule number fluctuation for the measurements.

## 3 RESULTS AND DISCUSSION

A conceptual framework for crowded and heterogeneous environments like biological cells and their cellular compartments is translational anomalous, subdiffusive motions of biomacromolecules due to geometric and/or energetic disorder [8]. The motions of proteins, RNAs or DNA can be hindered either by molecular crowding modeled by spatial, geometric restraints of fractals or by energetic landscapes/chemical binding represented by continuous-time random walks with heavy tails. This has been coined a coexistence of an ergodic anomalous diffusion and a nonergodic process of dynamics of macromolecules in living cells and their complex environment. Deciphering the molecular nature of the coexistence of ergodic anomalous process and nonergodic behavior constitutes an important quest of the field. 

### 3.1 Translational Anomalous Motion with Unbroken Ergodicity: Bridging Single-Molecule Approaches with Ensemble Averages

We first develop analytical models for the simulation of 3D subdiffusive motion. Widely used experimental methods such as fluorescence fluctuation spectroscopy and imaging like fluorescence correlation spectroscopy reveal only apparent values of the diffusion coefficients as well as α values. As shown in Table **2**, the developed fractal generators yield realistic scaling exponents α and $\Gamma_{\alpha }$ values of the 3D random walks on the their structures. Molecular crowding means that the possibilities of molecule motion are geometrically restricted by the local cellular environment where the molecule motion takes place. The less the exponent α the higher the geometric crowding of the underlying structure of motion, which can be quantified by the ratio of *d_f_*/*d_w_* as depicted in Fig. (**5**). The ratio is specific for the type of generator used.

**Fig. (6):**
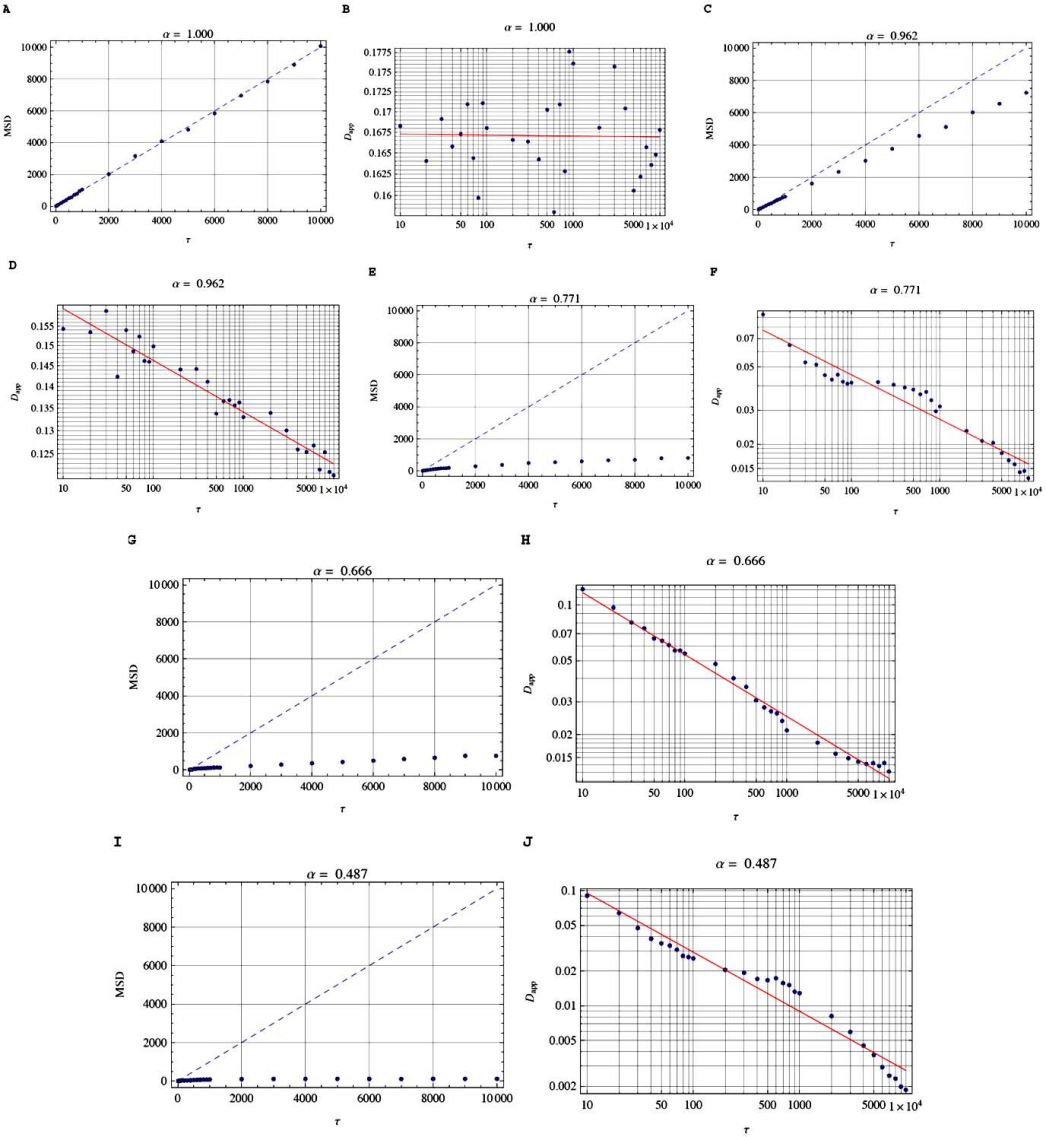
Linear plots of simulated MSD(*τ*) versus *τ* (panels **A**, **C**, **E**, **G**, **I**) and corresponding double-logarithmic plots of simulated apparent diffusion coefficients *D*_app_ versus *τ* (panels **B**, **D**, **F**, **H**, **J**) for different values of the simulated anomalous exponent α. The temporal autocorrelation defines the time scale *τ* of diffusion. In FCS, the autocorrelation is the crosscorrelation of the fluorescence signal with itself and is obtained by comparing a measured value at a time *t* with that at a time delayed by *τ*. *τ* is given in time steps covering a range from about 10 µ s to 100 ms. For all simulated α values, we strictly observe compliance with the scaling law of the simulated apparent diffusion coefficient *D*_app_ = 0.153∙ 10^-10^ [*m*^2^/s] of the fast mobile GFP-tagged RPA subunit in living HeLa cell over at least 3 orders of magnitudes of the time scale *τ*. The very small *τ* values are below the experimentally accessible time scale because of the diffraction limit of the confocal optics used.

For a confocal observation volume ΔV of 0.14 fL (1 fL = 10^-15^ L) with a radial waist *ω_x-y_* = 196 nm of the excitation laser and a half-length of *ω_z_* = 0.478 nm, we measured an average diffusion time *τ*_*D*,1_ = 0.628 ms over all time scales *τ* across this 3D observation volume ΔV for the fast mobile GFP-tagged RPA subunit in living HeLa cell by fixing *α* = 1 [14]. The corresponding average apparent translational diffusion coefficient for the fast mobile fraction was *D*_app_ = 0.153 ∙ 10^-10^ [*m*^2^/s]. The measurements were performed with one-photon FCS [14]. In Fig. 6, we strictly observe compliance with the scaling laws of the simulated apparent diffusion coefficient *D*_app_ = 0.153 ∙ 10^-10^ [*m*^2^/s] for all simulated α values over at least 3 orders of magnitudes of time scale *τ*. The very small *τ* values are below the experimentally accessible time scale because of the diffraction limit of the confocal optics used. As shown in Fig. (**6A, C, E, G, I**), the linear plots of simulated MSD(*τ*) versus *τ* clearly reveal translational anomalous motion for α ≠ 1. For better comparison, normal diffusive motion is indicated by dashed lines. In Fig. (**6B, D, F, H, J**), the apparent diffusion coefficients *D*_app_(*τ*) are plotted as lines from the double-logarithmic plot of the simulated MSD(*τ*) versus *τ* by means of Eqn. (3). Those lines give the scaling law of the measured *D*_app_ for different α values. Since in real experiments there are only a limited number of data points *D*_app_(*τ*) available, we verify the approximation for which each single value of *D*_app_ is found at time τ from the slope of the straight line between the origin and MSD(τ) [9]; this slope is 6 ∙ *D*_app_(τ) and the approximated *D*_app_ values are given as dots in Fig. (**6B, D, F, H, J**). We obtain good agreement between the approximated *D*_app_ values (dots) and the exact *theoretical* scaling laws (solid lines) in Fig. (**6B, D, F, H, J)**.

In Table **3**, simulated data are given to account for the diffusion time *τ*_*D*,2_(τ), e.g. of the slow mobile fraction of GFP-tagged RPA subunit in living HeLa cells [14], at different time scales τ. The average diffusion time *τ*_D,2_ = 5.340 ms over all time scales τ was measured across the 3D observation volume ΔV of 0.14 fL by fixing α = 1 [14]. The measured value matches the expectation. Table **3** demonstrates that the diffusion time decreases for smaller values of a α ≠ 1 but increases for a larger time scale *τ* at a given value of a α ≠ 1. The effect of translational anomalous motion is substantially greater if α differs much from 1. An α value close to 1 contributes little to the time dependence of subdiffusive motion. Thus, it is important to measure the anomalous dynamics on different time scales *τ* and to couple the analysis of how experimental parameters change with predictions from mechanistic models.

So far we considered only one diffusing component. Now, we shall examine to what degree of accuracy translational anomalous motion can be distinguished in two-component systems. Usually, the measured diffusion time *τ_D_*, the photon count rate detected from each kind of molecules, the relative quantum yield difference, and the molar concentration of each component in dilute solution are found by fitting an autocorrelation function for two different components to the experimental data [31]. In a situation of two labeled components in dilute solution, e.g. fast and slow mobile fractions, the measured diffusion time should differ at least by a factor of 2. A difference by a factor of 2 in diffusion times corresponds, for ideal spherical molecules, to a mass ratio of 1:8. Under certain conditions in dilute solution, a ratio of 1:5, e.g. for DNA/protein interactions, may be sufficient. In well-defined systems of dilute solution, differences in diffusion time down to 1:1.6 can be detected [31]. For globular molecules freely diffusing in dilute solution, this corresponds to a mass ratio of labeled ligand to formed complex or, generally speaking, of fast and slow mobile fractions of 1:4. Unfortunately, in a crowded environment of a living cell or its nucleus the motion of molecules are complicated as shown in Table **3** and the correlation data will be difficult to interpret. We present a criterion that rules out differences in diffusion times of two components which cannot be resolved in the correlation analysis of fluorescence fluctuation time series (photon streams) by FCS or FCCS or time-dependent fluorescence imaging under anomalous, subdiffusive motion. Assume that there is a fixed ratio of two diffusion times *τ*_*D*,1_ ≤ *τ*_*D*,2_ corresponding to the masses of two components with *m*_1_ ≤ *m*_2_ . If the resolution limit of *τ*_*D*,2_ to *τ*_*D*,1_ in the auto- or crosscorrelation function is given by, for example, $ℵ=4^{1/3}≃1.6$ for comparable quantum yields of the two components, high fluorescence signal (number of photons detected for each species) and a molar fraction of the formed slower species that should not fall below 0.5 as has been shown by experiments and simulations under normal diffusive motion in dilute solution [31], the following holds

(15) $$\frac{\tau_{D,2}}{\tau_{D,1}}\geq ℵ.$$

Our Eqn. (15) guarantees that *τ*_*D*,1_ ≤ *τ*_*D*,2_ . In the case of two-color crosscorrelation or/and two-color fluorescence imaging (co-localization experiments), the second component is also a two-color species *gr*, for example a different molecular complex *gr* with an additional ligand. If we use this relation and additionally assume that *τ*_*D*,2_ = *τ*_*D*,1_ + Δ*τ_D_* with Δ*τ_D_* the deviation from *τ*_*D*,1_ satisfying our previous assumption of the resolution limit Eqn. (15), we then first derive a relation, which contains *τ*_*D*,2_ only and the deviation Δ*τ_D_* corresponding to a resolution limit for two different molecule species (two components)

(16) $$\tau_{D,2}-\frac{ℵ}{ℵ-1}\cdot \Delta \tau_{D}\leq 0.$$

**Fig. (7):**
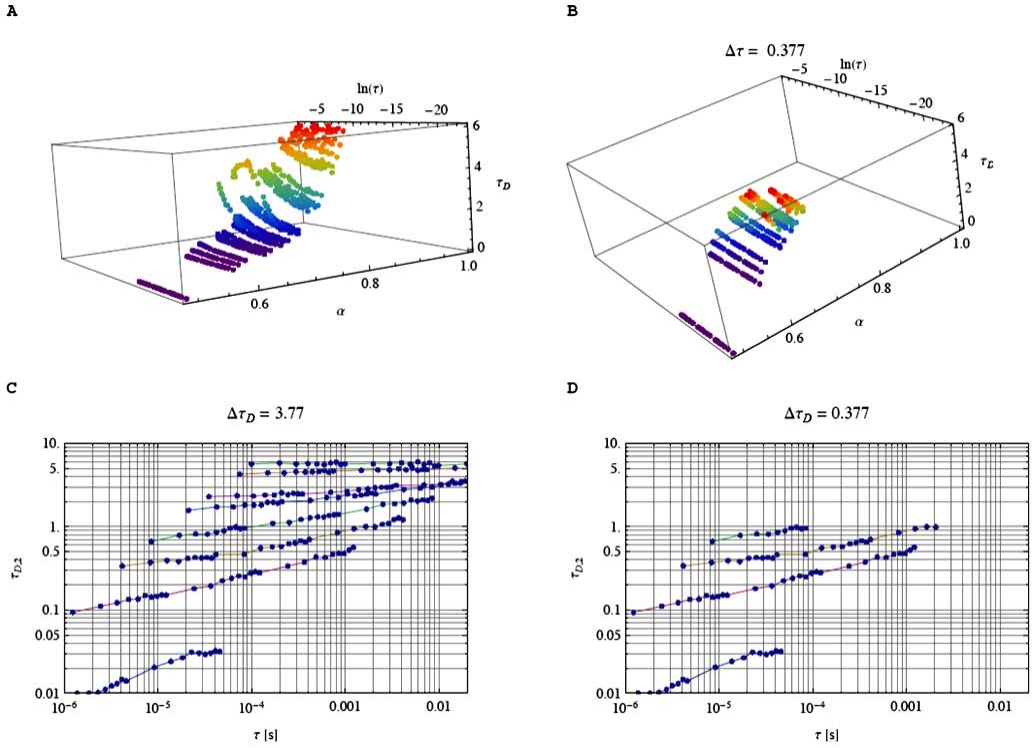
The crowding exponent α determines the resolution of differences in diffusion times in addition to photophyscial parameters well-known for normal motion in dilute solution. Panel **A**: Total set of simulated diffusion times as function of *τ* and α. The graph contains the behavior of diffusion times for some of the generators. It is obvious that *τ_D_* decays with decreasing α and increases due to the scaling law *τ^α^* in the direction of *τ*. Panel **B**: Subset of simulated diffusion times *τ_D_* satisfying the relation *τ_D_*_,2_ ≥ אּ.*τ_D_*_,2_. Here, we choose $ℵ=4^{1/3}≃1.6$. Panel C: Total set of diffusion times *τ_D_*_,2_ as function of *τ* and α. Simulated α values from top to bottom: 1.0, 0.977, 0.924, 0.884, 0.829, 0.800, 0.745, 0.640. Panel D: This subset of values *τ_D_*_,2_ = *τ_D_*_,1_ + Δ*τ* shows that for anomalous diffusive motion caused by molecular crowding the resolution limit given by the mass ratio *τ_D_*_,2_/*τ_D_*_,1_ ≥ אּ is not anymore guaranteed over all time scales *τ*. Simulated α values satisfying the chosen mass relation $ℵ=4^{1/3}≃1.6$ from top to bottom: 0.829, 0.800, 0.745, 0.640.

Our Eqn. (16) is valid for ${\Delta \tau }_{D}^{\min}\leq {\Delta \tau }_{D}\leq {\Delta \tau }_{D}^{\max}$ . Here ${\Delta \tau }_{D}^{\min}=\tau_{D,2}\cdot \left(ℵ-1\right)/ℵ$ and ${\Delta \tau }_{D}^{\max}=\tau_{D,2}^{\max}\cdot \left(ℵ-1\right)/ℵ$, where $\tau_{D,2}^{\max}$ is the maximal value of the diffusion times observed in the set. This criterion allows us to select those values of *τ*_*D*,2_ , which satisfy Eqn. (15) in connection with the independent variables of the time scale *τ* and the anomalous exponent *α* (see Fig. 7, left panels). If we now change Δ*τ* we observe that only a fraction of the available values for *τ*_*D*,2_ will satisfy relation (15) for Δ*τ* in a certain range. The selection of such resolution range in *τ*_*D*,2_ according to the mass ratio $ℵ=4^{1/3}≃1.6$ reported for normal diffusive motion in dilute solution under well-defined and optimized experimental (optical) setup conditions [31] is shown in Fig. 7 (right panels). These simulations demonstrate that the ability to distinguish between two different molecular species in a crowded environment depends strongly on the time scale *τ* at which the subdiffusive motion occurs and on the degree of crowding quantitatively expressed by the *α* value. Under anomalous subdiffusive motion due to molecular crowding, the specified value of the difference in diffusion times of two components Δ*τ_D_* by a factor of 1.6 at their mass ratio of 1:4 measured for normal diffusive motion under well-defined and optimized experimental conditions at comparable quantum yields for both components, a molar fraction of 0.5 and a high fluorescence signal [31] does not anymore guarantee that the two components can be resolved at all time scales *τ* of the measurement and at all *α* values for *α* ≠ 1.

In Fig. (**7**), we presented our simulated data for the resolution limit of two fractions with different molecular masses. We have first demonstrated that the crowding exponent α determines the resolution of differences in diffusion times in addition to the photophyscial parameters well-known for normal motion in dilute solution. For optimization of quantum yields of two components, number of photons detected for each species and the molar fraction of the formed slower species that should not fall below 0.5, etc., we refer to the experimental and simulated data available for normal diffusion in dilute solution [31]. Under optimized experimental conditions of the photophyscial parameters for two fluorescent components, we show that no better resolution of measured diffusion times can, in principle, be obtained than those given in Fig. (**7**) for a chosen $ℵ=4^{1/3}≃1.6$ . This is due to the crowding of the intracellular environment.

**Fig. (8):**
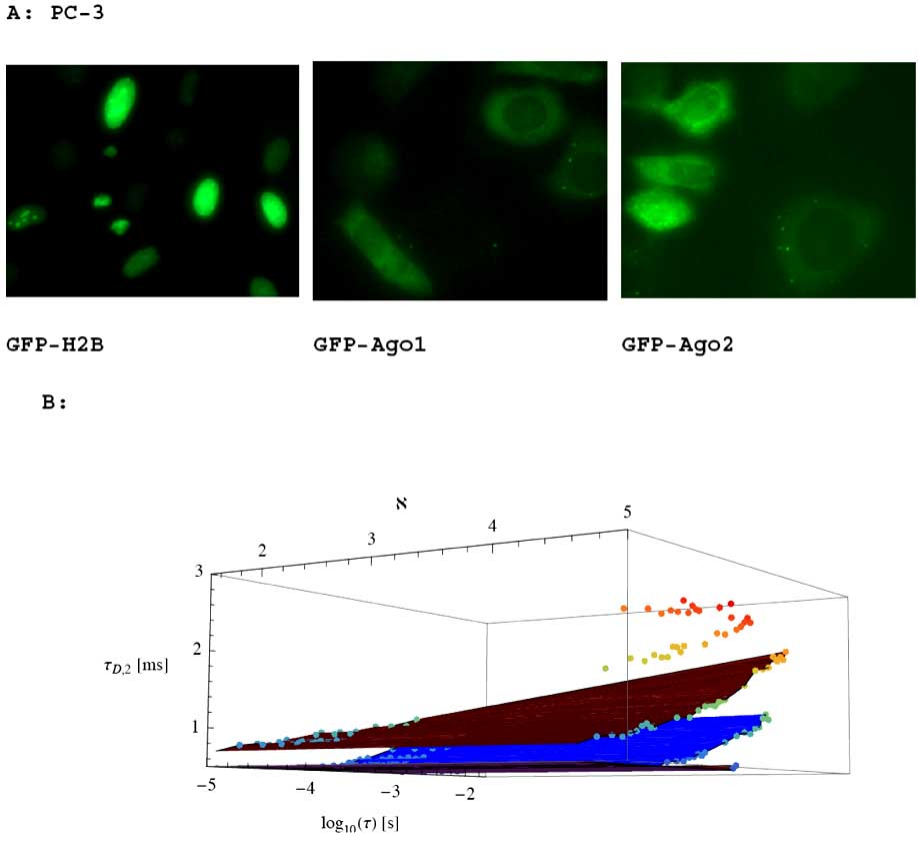
Fluorescence images of PC-3 cells transfected with GFP-Ago1 or GFP-Ago2 overexpression vector. A: The images were provided courtesy of Dr. Long-Cheng Li at the University of California, San Francisco. GFP-H2B served as a control for nuclear-specific localization. Although enrichment varied, GFP-Ago1 and GFP-Ago2 were visible in the cytoplasm and nucleus of prostate cancer cells. B: We demonstrate that GFP-Ago complexes located at target sites must fulfill the resolution criterion אּ given in Eqn. (15). Simulated diffusion times *τ_D_*_,2_ for GFP-Ago located at the target sequence (second component, GFP-Ago-Complex) in the presence of GFP-Ago (first component) of living cells for different α values and chosen mass relations $ℵ=4^{1/3}≃1.6$, $ℵ=6^{1/3}≃1.8$, $ℵ=8^{1/3}≃2$, $ℵ=10^{1/3}≃2.15$, $ℵ=100^{1/3}≃4.64$ between *putative* GFP-Ago-Complex (second component) and GFP-Ago (first component). Simulated anomalous exponent α , from top to bottom: 1.0,0.977,0.924,0.884,0.829,0.800. Measured values for o = 1 are in good agreement with known data of molecular weights of GFP-Ago2 and GFP-Ago1. The data points are from our theoretical predictions.

The argonaute (Ago) family of proteins are key regulators of RNA interference (RNAi) and RNA activation (RNAa) that function, in part, by recruiting small duplex RNAs [32-39]. It is well known that duplex RNAs [termed small interfering RNAs (siRNAs)] guide Ago proteins to complementary transcripts in the cytoplasm of cells to trigger RNAi [36-39]. Duplex RNAs [termed small activating RNAs (saRNAs)] can also activate gene transcription in the nucleus of cells to facilitate RNAa [32-35]. However, the mechanism of RNAa is complex and evidence is limited demonstrating physical interactions of Ago proteins with targeted nuclear sequences. We first apply our theoretical predictions under conditions of unbroken ergodicity to the time resolutions of molecular complexes formed in human prostate cancer cells (PC-3) transfected with fusions of green fluorescence protein (GFP) and argonaute proteins (Ago1, Ago2) to determine the lower limit of time resolution according to mass differences. Fluorescence images show cellular distribution of GFP-Ago1 and GFP-Ago2 following overexpression in PC-3 cells (Fig 8A). Note GFP-Ago1 and GFP-Ago2 can be found in both nuclear and cytoplasmic compartments of cells. GFP-fusion protein GFP-H2B was also transfected into PC-3 cells as a control for nuclear-specific localization; H2B is a core histone protein that functions exclusively in the nucleus (Fig **8A**). We believe that diffusive motions of any GFP-Ago protein (free GFP-Ago) bound at targeted sequence (GFP-Ago-Complex, bound GFP-Ago) can be time-resolved in living cells and their compartments if they fulfill the theoretical resolution limit (Fig. 8B). The molecular weights of both Ago1 and Ago2 are about 100 kDa, while the molecular weight of GFP is 27 kDa. The measured apparent diffusion coefficient of GFP in living cells is around 0.275 ∙ 10^-10^[*m*^2^/s}. Based on these experimental values, we simulate the diffusion times and lower limits for the time resolution of two components in the fluorescence images. In the examples so far chosen, good agreement between the measured apparent diffusion coefficients at α = 1 and the known molecular weights show that our predictions are reasonable. In Fig. (**8B**), we present the simulated diffusion times *τ*_*D*,2_ for a GFP-Ago located at the target sequence (second component, bound GFP-Ago that is also called GFP-Ago-Complex) in the presence of 'free' GFP-Ago, i.e. unbound GFP-Ago (first component), for different simulated α values. Differences in diffusion times between the two components in a crowded environment can only be detected down to the אּ value for the chosen mass relation between GFP-Ago located at the target sequence (second component, bound GFP-Ago) in the presence of free GFP-Ago (first component) with $ℵ=4^{1/3}≃1.6$, $ℵ=6^{1/3}≃1.8$, $ℵ=8^{1/3}≃2$, $ℵ=10^{1/3}≃2.15$, $ℵ=100^{1/3}≃4.64$. It is apparent from Fig. (**8B**) that the diffusion times of the second compound, $\tau_{D,2}=\tau_{D,1}+\Delta \tau ;\Delta \tau =\tau_{D,1}\cdot \left(ℵ-1\right)$, which can just be resolved in fluctuation time traces, decreases with decreasing α. This is predicted by the theory. In stationary FCS, the time spent dwelling on a spot between *t *= 0 and *τ_D_* is partially wasted, since little new information is obtained in time intervals much smaller than the sample correlation time apart from overcoming photon statistics noise. This spare time can be effectively utilized by scanning into and observing new volumes, each of which are visited only occasionally. Single-molecule studies do not always use information from one individual molecule only or interpret every single molecule in the bulk phase [40]. Bridging single-molecule approaches with ensemble averages should yield interesting results [40, 41]. The results assume that uncorrelated photons are measured which means they are statistically independent. Because the experimental apparatus requires time intervals of several milliseconds up to seconds and even longer, the photon correlations will be lost for times much longer than the coherence time that is the inverse of the bandwidth of the laser. This phenomenon can be understood by noting that if the simulation/measurement time *T* is very large, many fluctuations take place, and hence we measure an average value of the fluctuations by imaging and spectroscopy and not the fluctuation itself. The longer the time interval *T*, the closer the measured value approaches the mean value. As a consequence, the measured statistics approaches the uncorrelated Poisson distribution. We provided a direct test of single-molecule trajectories in solution-phase by means of fluorescence fluctuation microscopy [10]. Our analysis moves beyond unphysical assumptions of diffusive measurements by time-dependent fluorescence fluctuation microscopy.

The currently well accepted approach to measure a single molecule as it flows through a well-defined probe/ observation volume is often not true single molecule [42]. Although there is only one analyte molecule in the observation volume during the measurement, poor signal-to-noise requires that bursts from many analyte species must be averaged in order to achieve a reasonable signal-to-noise ratio. This makes it difficult to distinguish between rare confirmers with a strong signal that occasionally pass through the observation volume (or confirmers in dynamic equilibrium) from a mixture of stable confirmers (Richard. A. Keller, personal communication, Los Alamos); molecules that travel fast get there first and have less time to diffuse and the diffusion width is small. In very dilute solutions without flow, with very high probability the first molecule to enter the observation volume is the molecule that just left. The reentry time depends on the size of the observation volume, the diffusion coefficient and the molar bulk concentration of other molecules of the same kind that are not the original molecule [43]. Fluorescence molecule counting is, strictly speaking, a method to determine the experimental single-molecule regime for measuring just one individual molecule at a time [10]. It provides the theoretical and experimental criteria to verify not only the average molecule number but rather the molecular number of independently distributed molecules in dilute solution [10] as well as in crowded and highly heterogeneous living cells and their compartments (Baumann, Foldes-Papp, 2010, in preparation) [44].

### 3.2 Translational Anomalous Motion with Broken Ergodicity

Single biomacromolecules in *in vitro* systems measured by immobilization on cover slips display a heterogeneous molecular behavior. These are studies on one and the same single molecule, i. e. the selfsame molecule, over the whole measurement time *T* [42, 43]. However, we consider non-immobilized molecules [10, 43]. We present here the simulation results of translational subdiffusive motion under crowded condition where the time step becomes now a Levy distribution of waiting times [8]. To fulfill the experimental single-molecule regime the molecule number *N* per 3D observation volume ΔV has to be adjusted to *N* << 1 [10, 43], and the single-molecule detection regime comes close to the average *N* = 0.048 and *N* = 0.0057 molecules and particles, respectively, per ΔV [45]. However, this experimental condition does not guarantee that one and the same molecule, i.e. the selfsame molecule, is measured over the whole measurement time *T* [10, 43, 46, 47]. A mathematical approach that rigorously solves the problem and produces the meaningful time for studying just one single molecule without immobilization or hydrodynamic/ electrokinetic focusing in solution and living cells is the meaningful-time concept first given in ref. [43, 46, 47]. These exact physical equations capture the reentry time of the selfsame molecule into the observation volume ΔV and depend on the size of the observation volume ΔV, the diffusion coefficient and the molar bulk concentration of other molecules of the same kind that are not the original molecule [43, 46, 47]. For the present results, we assume that the selfsame molecule is observed. Fig. (**9**) shows three examples for simulated data of the selfsame molecule from which MSD and the power law behavior can be evaluated. Random walks are usually carried out on regular lattices, which have the disadvantage that the spatial and temporal steps are correlated. However, randomness in cellular systems can be either spatial (anomalous) or temporal (heterogeneous). In order to separate both processes we introduced in the previous sections anomalous random walks on fractals that represented crowded environments. In order to mimic heterogeneous behavior in crowded environments of living cells, we need to introduce so-called continuous time random walks (CTRW). CTRWs were originally performed on regular lattices [48]. In this section, we combine both ideas and perform random walks on fractals using CTRWs [49]. This purely stochastic molecule behavior leads to subdiffusive motion with broken ergodicity in our simulations.

In a CTRW, the molecule has to wait for a time *t* on each site of the fractal before performing the next step. This waiting time is a random variable independently chosen at each new step according to a continuous distribution *Ψ* (*τ* ). In our case, a stable Levy distribution with Levy index 3/2 is used for the waiting time steps $\psi \left(\tau \right)=e^{-1/\left(2\tau \right)}/\left(\sqrt{2\pi }\cdot \tau^{3/2}\right)$ . The first and second moments of this distribution do not exist. We assume that the molecule can step to one of the nearest neighbors generated by the fractal generator. In addition, *Ψ* (*τ* ) is independent of the location of the molecule, i.e. we decouple spatial and temporal coordinates. One can think of this process as a diffusive motion among traps, but the trapping time will change for each site on the lattice if this site is visited again during the random walk. If we perform a walk on a regular lattice, disorder can be mimicked through waiting time distributions *Ψ* (*τ* ) displaying long-time tails

(17) $$\psi \left(\tau \right)∼\tau^{-\left(1+\gamma \right)}\mathrm{with}\;0\;≺\gamma ≺1.$$

The probability to perform *n* steps during time *τ* is denoted by *χ_n_τ* , which is related to the waiting time distribution by the Laplace transform

(18) $$L\left(\chi_{n}\left(\tau \right)\right)=\chi_{n}\left(s\right)={\left(\psi \left(s\right)\right)}^{n}\cdot \left(1-\psi \left(s\right)\right)/s.$$

This probability is needed to analyze the MSD for a random walk on a fractal support carried out as a CTRW [49, 50]. The MSD traveled by the molecule during the time τ is given by

(19) $$\langle {\vec{r}}^{2}\left(\tau \right)\rangle =\sum_{n=0}^{\infty }\langle {\vec{r}}_{n}^{2}\rangle \chi_{n}\left(\tau \right),$$

where $\langle {\vec{r}}_{n}^{2}\rangle$ is the average distance traveled in *n* steps on the fractal. For fractals, we found that $\langle {\vec{r}}_{n}^{2}\rangle ∼n^{\alpha }$ so that after carrying out the summation in the continuous limit the asymptotic behavior $\langle {\vec{r}}_{n}^{2}\rangle$ is found to be

(20) $$\langle {\vec{r}}^{2}\left(\tau \right)\rangle ∼\tau^{\alpha \cdot \gamma }=\tau^{\tilde{\gamma }}.$$

We observe in our simulations that, contrary to what is expected from random walk on fractal support (RWF), the temporal averaged MSD_*T*_ Eqn. (10) leads to a simple diffusive behavior with scaling exponents that strongly differ from one track to another. This distribution of scaling exponents renders a system inhomogeneous: an ensemble of simple diffusers with different diffusion coefficients. Since α and $\tilde{\gamma }$ satisfy $0≺\alpha ,\tilde{\gamma }≺1$, so does the product $\alpha \cdot \tilde{\gamma }$ and the diffusive motion is thus subdiffusive. The main point here is that the ensemble average and the time average do not deliver the same scaling exponent anymore, i.e. we have broken ergodicity.

Since the experimental conditions to measure a selfsame molecule over an extended period of time, at which biology is taken place, in living cells or even in dilute solution-phase are very restrictive [43, 46, 47], we need to perform the time average over a subpopulation of different single molecules of the same kind:

(21) $${\langle {\langle {\vec{r}}^{2}\left(\tau \right)\rangle }_{T}\rangle }_{\mathrm{sub}-\mathrm{ens}}={\langle {\langle {\vec{r}}^{2}\left(\tau \right)\rangle }_{\mathrm{sub}-\mathrm{ens}}\rangle }_{T}={\langle {\langle n^{\tilde{\gamma }}\left(\tau \right)\rangle }_{\mathrm{sub}-\mathrm{ens}}\rangle }_{T}\cdot$$

Our experimental single-molecule regime given by Eqn. (21) differs from averaging over the whole molecule ensemble suggested by Meroz, Sokolov, Klafter (2010) [8]. For time averages over subpopulations of single molecules of the same kind, the temporal auto- and crosscorrelation functions are found for the first time

(22) $$G\left(\tau \right)=\frac{1}{N}\cdot {\left(1+{\left(\tau /\tau_{D}\right)}^{\tilde{\gamma }}\right)}^{-1}\cdot {\left(1+\left(1/s^{2}\right)\cdot {\left(\tau /\tau_{D}\right)}^{\tilde{\gamma }}\right)}^{-\left(\dim-2\right)/2}+\mathrm{DC},$$

(23) $$\tilde{\gamma }=\alpha \cdot \gamma .$$

Here, *τ_D_* is the diffusion time and *τ*_*D*,gr_ is the two-color cross-correlated diffusion time given by Eqn. (2). s is the so-called structural factor defined as *s* = *ω_z_*/*ω_x-y_* . In the case of 3D diffusive measurements, dim = 3. For 2D diffusive measurements (e.g., in membranes), dim = 2. DC is the limiting value of *G(τ)* for *τ* → ∞, which is normally 1. By fitting Eqn. (22) to the time traces that are recorded for the subpopulation of single molecules of the same kind without interacting partner, e.g. without ligand, in the crowded environment of a living cell and their cellular compartments, respectively, $\tilde{\gamma }=\alpha$ is obtained. Knowing the crowding parameter α for the cell type and cellular compartment type, the heterogeneous parameter γ can be extracted from the measurements in the presence of the interacting reaction partner, e.g. ligand, for the same $\alpha \cdot \tilde{\gamma }$ (Eqn. (23)) is not a simple fitting parameter in the temporal auto- and two-color crosscorrelation functions (Eqn. (22) and Eqn. (2)) since it is related via Eqn. (23) to the physical model given in Eqn. (19) for our proposed Eqn. (21). We already derived an analytical solution for $\tilde{\gamma }$ in the special case of *γ* = 3/2 [44].

## CONCLUSIONS

The generally applied assumption of normal Brownian motion of macromolecules works well for *in vitro* experiments under dilute conditions, but does not apply for a crowded environment of living cells and their cellular compartment, e.g. the nucleus. Fixing α = 1 in auto- and crosscorrelation functions for one and two species (components) results only in a coarse average over all measured time scales *τ* of subdiffusive motion. Our simulations show that determination of molecular weights from measured diffusion times and apparent diffusion coefficients, respectively, in temporal auto- and two-color crosscorrelation analyses as well as from fluorescence imaging data are difficult to interpret and biased in crowded environments of living cells and their cellular compartments unless the anomalous dynamics on different time scales *τ* is coupled with the analysis of how experimental parameters change with predictions from simulated diffusion times and apparent diffusion coefficients, respectively, and mechanistic models.

The basic property of ergodic systems is that the time average and the ensemble average on the tracks result in the same MSD. Nonergodicity is given if the time average and the ensemble average on the tracks are different. The nonergodic situation is strictly valid only for the selfsame molecule. A mathematical approach that produces the meaningful time for studying the selfsame molecule (just one single molecule) without immobilization or hydrodynamic/ electrokinetic focusing in solution and living cells is the meaning-ful-time concept first derived in ref. [43, 46, 47]. These exact physical equations capture the reentry time of the selfsame molecule in the observation volume ΔV and depend on the size of the observation volume ΔV, the diffusion coefficient and the molar bulk concentration of other molecules of the same kind that are not the original molecule [43, 46, 47]. Since the experimental conditions to measure a selfsame molecule over an extended period of time, at which biology is taken place, in living cells or even in solutions are very restrictive, we need to perform the time average over a subpopulation of different single molecules. The temporal auto- and crosscorrelation functions are found for the first time with the crowding parameter α for the cell type and cellular compartment type, respectively, and the heterogeneous parameter γ that can be obtained from the measurements in the presence of the interacting reaction partner, e.g. ligand, with the same α value. The product $\tilde{\gamma }$ of the crowding parameter α and the heterogeneous parameter γ is not a simple fitting parameter in the temporal auto- and two-color crosscorrelation functions because it is related to the proper physical models of anomalous (spatial) and heterogeneous (temporal) randomness in cellular systems.

This work extends previous work on trafficking of mature miRNA-122 into the nucleus of living liver cells, where we first presented FCS/FCCS-artifact free subdiffusive measurements of the formed fluorescent complex in the cytoplasm and nucleus by two-photon imaging [51]. A similar biological interpretation of miRNA traveling into the nucleus was proposed independently by Ohrt *et al.* [13, 52] from quantitative two-color FCCS measurements and respective calculations of molecular weights in living cells but without taking into account the difficulties and artifacts in the proper interpretation of quantitative subdiffusive measurements that we presented above. In crowded environment of living cells and their compartments, diffusive motion is characterized by the time-dependence of the mean-square displacement and the apparent diffusion coefficient; the measured diffusion time is not anymore a fixed constant at all time scales *τ* for a given size of the observation volume. Here, we first show that plausible biological mechanisms from FCS/FCCS and fluorescence imaging data are highly questionable without proper quantitative physical models of subdiffusive motion and temporal randomness. At best, such quantitative FCS/FCCS and fluorescence imaging data are difficult to interpret in living cells under crowding and heterogeneous conditions. It is challenging to translate proper physical models of anomalous (spatial) and heterogeneous (temporal) randomness in living cells and their cellular compartments like the nucleus into biological models of the cell biological process under study testable by single-molecule approaches in living cells. Otherwise, quantitative FCS/FCCS and time-dependent fluorescence imaging measurements in living cells are not well described and cannot be interpreted in a meaningful way.

## Figures and Tables

**Fig. (2):**
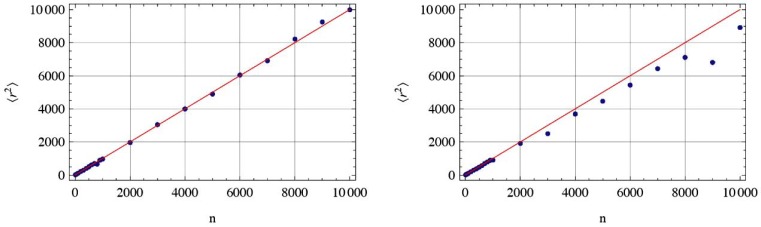
The mean-square displacement of a normal random walk (RW) on a 2D regular square grid (left panel) and the MSD of a 3D random walk on a generalized Sierpinski carpet (right panel). For large track lengths *n*, it is obvious that the linear behavior $\langle {\vec{r}}^{2}\rangle ∼n$ changes to the anomalous $\langle {\vec{r}}^{2}\rangle ∼n^{\alpha }$ . The case shown with *α* = 0.98 was calculated for a generalized Sierpinski carpet. The number of tracks used to derive the MSD are in both cases *M* =10_3_ . In both plots the straight line represents the linear behavior. For anomalous diffusion, a significant deviation corresponding to the law *n^α^* is obvious.

**Fig. (3):**
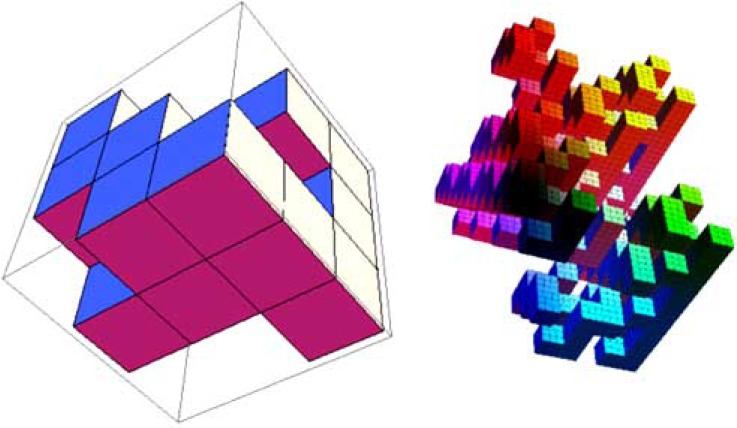
Construction of a generalized Sierpinski carpet with ν = 3 and µ = 15 (12 empty sites of deleted cubes). The right graph shows part of the 4th iteration of the fractal support. In the simulations of experimental data we actually used the 12th iteration of the fractal which is not shown here due to space requirements.

**Fig. (4):**
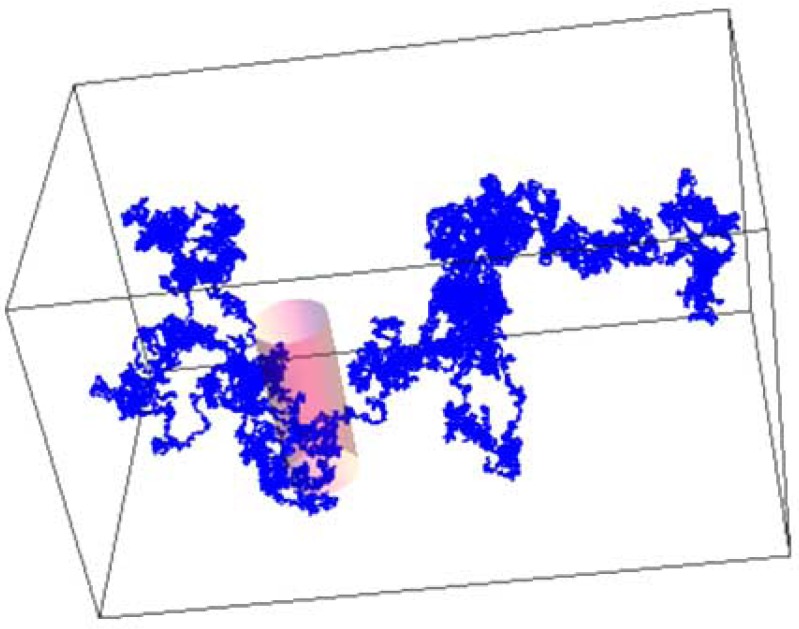
The measurement system: 3D translational anomalous diffusive motion within the observation volume ΔV = 0.14 fL (in pink color). Simulation steps n=10000.

**Fig. (5):**
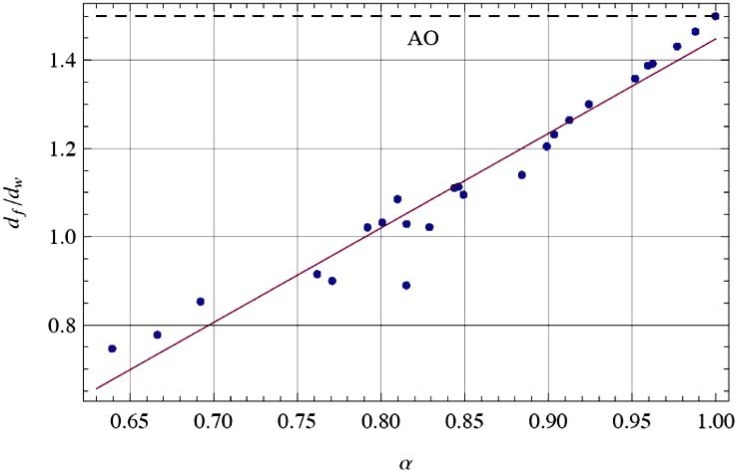
Relation between the fractal ratio *d_f_/d_w_* as a function of α . The fixed ratio of Alexander and Orbach (AO) with *d_f_/d_w_* = 3/2 is also shown as a dashed line (top of the graph) [18]. The AO relation is satisfied for the limit of a Brownian random walk with *α* =1 but shows a large deviation for fractional Brownian walks on GSC. *d_w_* characterizes the *dynamic* process of diffusive motion and *d_f_* describes the *static* geometrical scaling of the mass of the molecule. Thus, the ratio *d_f_/d_w_* is a measure of molecular crowding.

**Fig. (9):**
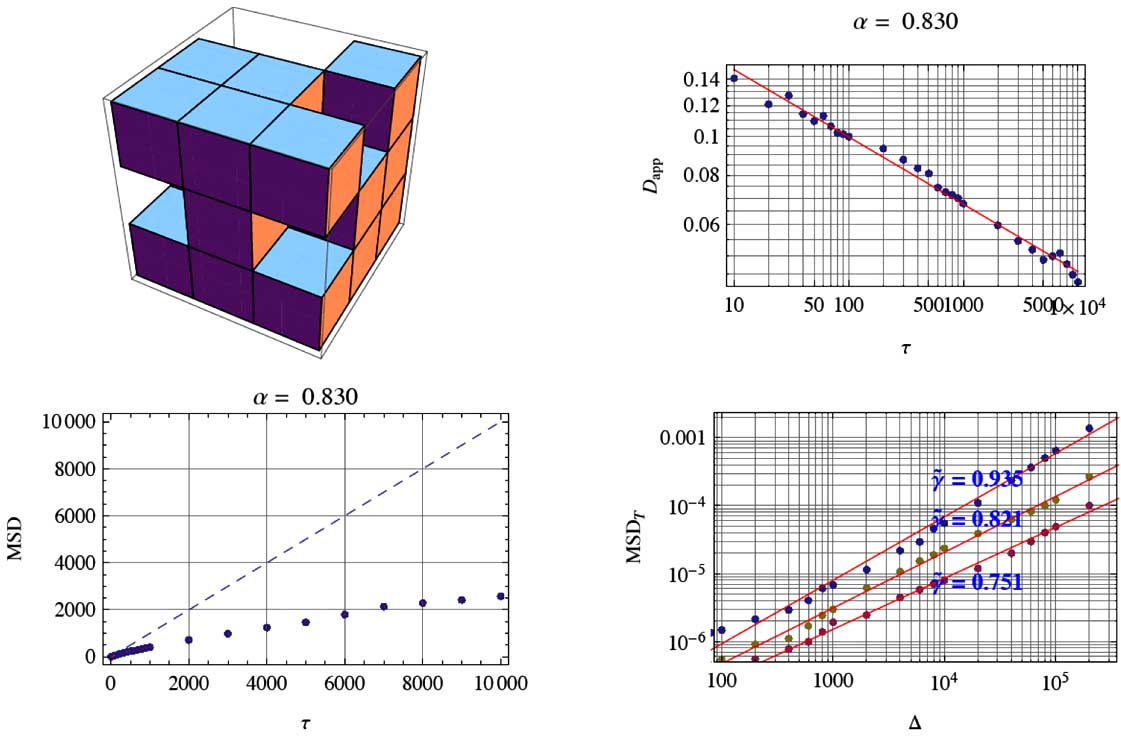
MSD for a specific generator of the fractal grid (top left). Top right the apparent diffusion coefficient is shown for the random walk on fractal support (RWF). For each track 10^4^ steps on the grid were performed in a random walk on fractal support and in a CTRW on the fractal support. The ensemble for the RWF consist of 10^4^ tracks from which the MSD follows (bottom left). For the CTRW the different tracks all have the same number of steps. The CTRW with the selected fractal generator shows a broad distribution of fractal dimensions. However, the ensemble mean for the same fractal generator shows a single reproducible fractal dimension of α = 0.830. The specific fractal dimensions following from the temporal averaging in the MSD are $\tilde{\gamma }=\left\{0.751,0.821,0.935\right\}$ shown in the lower right figure. Since the RWF scaling exponent is fixed to α = 0.830 we can derive the time scaling from the relation $\tilde{\gamma }=\alpha \cdot \gamma$ to be *γ* = {0.905,0.989,1.126}. This behavior shows that for the MSD the ensembles average and the time average is not any more the same; i.e. we have broken ergodicity.

**Table 1.** Anomalous Diffusive (Subdiffusive) 3D Motion on 3 Fractal Supports. Type of Fractal: 3D Sierpinski Carpet. The Fractal Generators are Depicted in the Model. The Simulated 3D Random Walks on Each Fractal Support are Shown in the Right Panels. Simulation Steps *n* =10000 . Different α and $\Gamma_{\alpha }$ Values were Obtained from the Double-Logarithmic Plot of the Simulated MSD(*t*) versus *t* Generated from the Different Fractal Supports. For Spatial and Time Steps of Unity, the Following α and $\Gamma_{\alpha }$ were Simulated from Top to Bottom: α = { 0.863, 0.766, 0.770}, $\Gamma_{\alpha }$ = { 0.158, 0.355, 0.332}

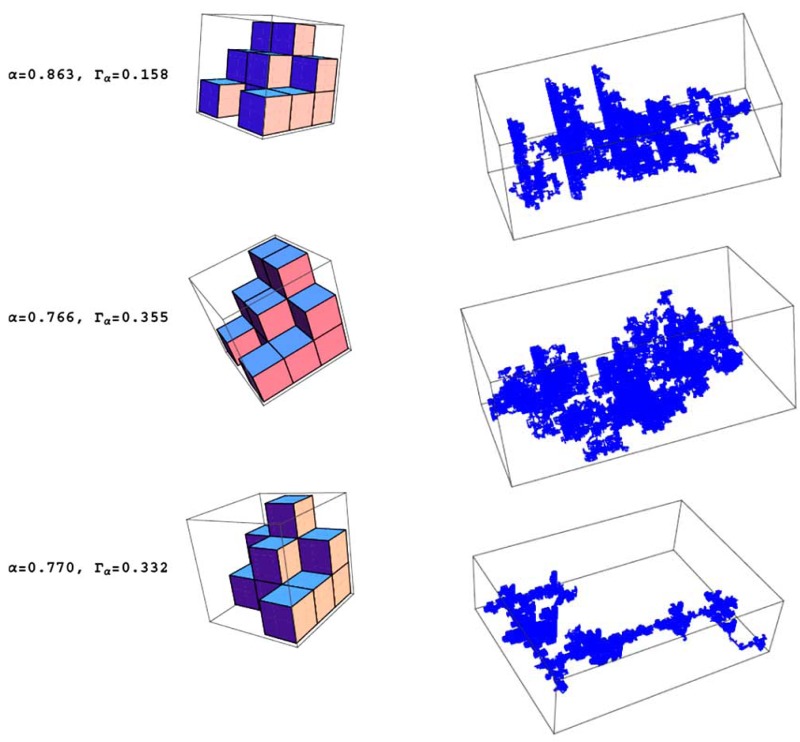

**Table 2.** Simulation of Values for α, $\Gamma_{\alpha }$ and Time Steps Δt with the Indicated Fractal Generators by Fixing the Spatial Step Lengths of the 3D Random Walks Δx to 10 nm on the Crowded Structure

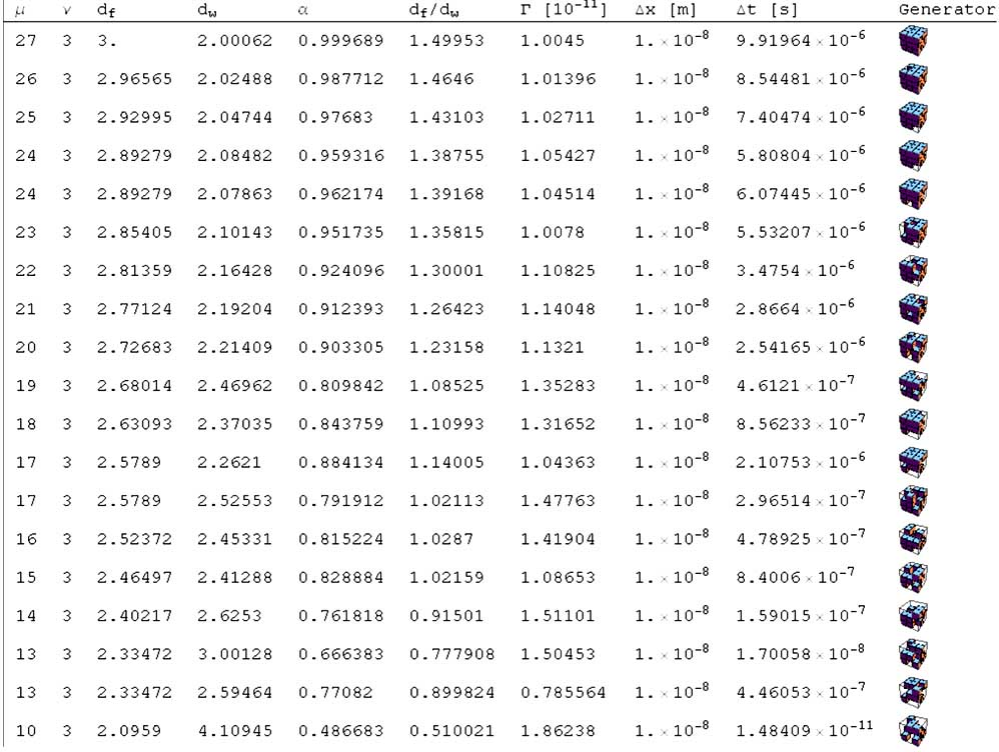

**Table 3.** Simulation of the Time Scale Behavior of the Slow Mobile Fraction of GFP-Tagged RPA Subunit in Living HeLa Cells. The Average Diffusion Time τ _D,2_(τ ) = 5.340 ms Over All Time Scales τ Across the 3D Observation Volume ΔV of 0.14 fL was Measured by Fixing α = 1 [14]. The Subdiffusive Motion in a Crowded Environment is Quantitatively Characterized by the Anomalous Exponent α. The Simulated Time Scale τ Covers a Time Range from about 10 µs to 100 ms at Which Diffusive Motion Occurred [14]

α	r_D,min_ [ms]	r_D,max_ [ms]
1.000	5.662	5.677
0.988	4.919	5.485
0.977	4.257	4.982
0.962	3.780	4.839
0.959	3.443	4.705
0.952	3.938	5.157
0.924	2.297	3.790
0.912	1.786	3.251
0.903	1.733	3.272
0.899	1.930	3.366
0.884	1.567	3.398
0.849	0.730	2.088
0.846	0.550	1.596
0.844	0.563	1.638
0.829	0.664	2.190
0.815	0.338	1.089
0.810	0.373	1.213
0.801	0.338	1.210
0.792	0.214	0.826
0.771	0.460	3.199
0.762	0.120	0.566
0.692	0.035	0.298
